# Mortality Attending the Operation of Tying the Large Arteries

**Published:** 1845-06

**Authors:** T. Inman

**Affiliations:** Liverpool


*Mortality attending the Operation of Tying the Large Arteries.* By T. Inman, of Liverpool.—Mr. Inman gives the following table, drawn up from a careful examination of all the medical periodicals for a long series of years.

| Artery subjected to ligature. | No. of Cases. | Deaths. | Proportion. |
|---|---|---|---|
| Cases collected by Phillips from the works of Boyer, Lancisi, Scarpa, Pelletan, &c. where old operation was performed, viz: | | | |
| Femoral tied, - - - | 22 | 6 | 1 in $3\frac{3}{4}$ |
| Humeral tied, - - - | 7 | 1 | 1 in 7 |
| Hunterian operation, | | | |
| Arteria innominata, - - | 6 | 6 | all died |
| Subclavian, - - - | 40 | 18 | 1 in 2 |
| Carotid, - - - - | 40 | 11 | 1 in 4 |
| Abdominal aorta, - - - | 3 | 3 | all died |
| Common iliac, - - - | 8 | 3 | 1 in $2\frac{2}{3}$ |
| Internal iliac, - - - | 4 | 2 | 1 in 2 |
| External iliac, - - - | 27 | 9 | 1 in 3 |
| Femoral, - - - | 42 | 7 | 1 in 6 |

Mr. Inman also gives the following table of the mortality attending the operation of hernia:—

| Where, or by whom recorded: | No. of Cases. | Deaths. | Proportion. |
|---|---|---|---|
| In Sir A. Cooper's work on Hernia, | 77 | 36 | 1 in 2 |
| By Travers, - - - - - | 14 | 8 | 1 in $1\frac{1}{2}$ |
| Dewar of Dunfermline, - - | 17 | 4 | 1 in 4 |
| Scarpa, (on Hernia), - - | 16 | 5 | 1 in 3 |
| Lawrence, (on Hernia), - - | 22 | 6 | 1 in 3 |
| Clement, - - | 8 | 3 | 1 in 2 |
| Hey, (he performed the operation 40 times, but no detailed account is given of all the cases,) | 12 | 6 | 1 in 2 |
| Wurtzburg from 1815 to 1842, | 56 | 24 | 1 in $2\frac{1}{2}$ |
| Recorded in different periodicals as isolated cases, | 88 | 30 | 1 in 3 |
| Malgaigne's,—hospitals in France,—Patients between 50 and 80 years, | 97 | 70 | 1 in $1\frac{1}{4}$ |
| all other ages, | 86 | 44 | 1 in 2 |
| Guy's Hospital, from September, 1841, to December, 1842, | 19 | 10 | 1 in 2 |
| Scotch hospitals during 1843, | 11 | 3 | 1 in $3\frac{2}{3}$ |
| Cases witnessed by author, | 6 | 3 | 1 in 2 |
| Liverpool Infirmary for two years, | 4 | 1 | 1 in 4 |
| Liverpool Northern Hosps. 9 years, | 12 | 6 | 1 in 2 |
| Total, | 545 | 260 | 1 in 2 |

*London Lancet.*

